# Construction and Immunogenicity of a Novel Multivalent Vaccine Prototype Based on Conserved Influenza Virus Antigens

**DOI:** 10.3390/vaccines8020197

**Published:** 2020-04-24

**Authors:** Anna Kirsteina, Inara Akopjana, Janis Bogans, Ilva Lieknina, Juris Jansons, Dace Skrastina, Tatjana Kazaka, Kaspars Tars, Irina Isakova-Sivak, Daria Mezhenskaya, Tatiana Kotomina, Victoria Matyushenko, Larisa Rudenko, Andris Kazaks

**Affiliations:** 1 Latvian Biomedical Research and Study Centre, LV-1067 Riga, Latvia; anna.kirsteina@biomed.lu.lv (A.K.); inara@biomed.lu.lv (I.A.); janis@biomed.lu.lv (J.B.); ilva@biomed.lu.lv (I.L.); jansons@biomed.lu.lv (J.J.); daceskr@biomed.lu.lv (D.S.); tatyanav@biomed.lu.lv (T.K.); kaspars@biomed.lu.lv (K.T.); 2 Department of Virology, Institute of Experimental Medicine, Saint Petersburg 197376, Russia; isakova.sivak@iemspb.ru (I.I.-S.); dasmez@iemspb.ru (D.M.); kotomina@iemspb.ru (T.K.); matyshenko@iemspb.ru (V.M.); rudenko.lg@iemspb.ru (L.R.)

**Keywords:** influenza, conserved antigens, hemagglutinin stalk, M2e protein, virus-like particles, protection, vaccines

## Abstract

Influenza, an acute, highly contagious respiratory disease, remains a significant threat to public health. More effective vaccination strategies aimed at inducing broad cross-protection not only against seasonal influenza variants, but also zoonotic and emerging pandemic influenza strains are urgently needed. A number of conserved protein targets to elicit such cross-protective immunity have been under investigation, with long alpha-helix (LAH) from hemagglutinin stalk and ectodomain of matrix protein 2 ion channel (M2e) being the most studied ones. Recently, we have reported the three-dimensional structure and some practical applications of LAH expressed in *Escherichia coli* system (referred to as tri-stalk protein). In the present study, we investigated the immunogenicity and efficacy of a panel of broadly protective influenza vaccine prototypes based on both influenza tri-stalk and triple M2e (3M2e) antigens integrated into phage AP205 virus-like particles (VLPs). While VLPs containing the 3M2e alone induced protection against standard homologous and heterologous virus challenge in mice, only the combination of both conserved influenza antigens into a single VLP fully protected mice from a high-dose homologous H1N1 influenza infection. We propose that a combination of genetic fusion and chemical coupling techniques to expose two different foreign influenza antigens on a single particle is a perspective approach for generation of a broadly-effective vaccine candidate that could protect against the constantly emerging influenza virus strains.

## 1. Introduction

Influenza, an acute, highly contagious respiratory disease, remains a significant threat to public health. The co-circulating influenza A (subtypes H1N1 and H3N2) and B (lineages Victoria and Yamagata) viruses cause seasonal epidemics, which affect a major part of the global population causing high morbidity and up to 645,832 influenza-associated deaths annually [1,2]. Consequently, seasonal influenza viruses are responsible for a significant economic burden on the health care systems and society [3,4].

Although influenza is considered to be a vaccine-preventable disease, the effectiveness of seasonal influenza vaccines varies greatly across the risk groups and seasons, on average between 19–60% [5,6]. Currently licensed vaccines induce a strain-specific antibody response, hence vaccine effectiveness is significantly compromised by the persistent antigenic changes of influenza viruses [7,8]. Continuous antibody-mediated immune pressure and the lack of a proof-reading function for RNA polymerase errors result in accumulation of point mutations in the hemagglutinin (HA) and neuraminidase genes and in constant emergence of mutant variants affecting immune recognition. This process, characteristic to both influenza A and B viruses, is known as antigenic drift and it has the potential to cause local outbreaks and seasonal epidemics [7,8,9,10]. Moreover, sporadic reassortment of segmented influenza A virus genomes in a double-infected host cell can result in an antigenic shift, which can lead to a global pandemic by producing an unpredicted virus strain to which the immunologically naive human population is particularly susceptible. Interspecies transmissions of influenza A viruses from animal reservoirs have resulted in four pandemics since the beginning of the 20th century; the Spanish influenza pandemic in 1918 is considered to be the most lethal in history, with a mortality rate of over 50 million cases [11,12].

Annual immunization with updated seasonal vaccine formulations is recommended [1], but prediction mismatches between the vaccine strains and circulating viruses can dramatically decrease the vaccine effectiveness [5,6,7,8,13]. Furthermore, the components of commercially available influenza vaccines are mainly obtained by propagating viruses in embryonated chicken eggs which is a complex, time-consuming, and expensive process with limitations for highly pathogenic avian-borne influenza virus vaccine preparations [14,15]. The capability to manufacture large amounts of strain-specific vaccine doses in the event of a sudden influenza pandemic is limited, which means that during the months of vaccine preparation, testing and distribution, society can be particularly vulnerable to the newly emerged virus strain [11,14,15]. The persistent threat of highly pathogenic poultry influenza viruses (H5Nx) and other zoonotic influenza strains (H6N1, H7N9, H10N8) overcoming the species barrier and adapting to mammalian hosts highlights a continuous pandemic risk [16,17,18,19]. Currently, numerous strategies to improve the effectiveness of seasonal influenza vaccines, like the use of new adjuvants, increased doses, or different vaccination strategies, are under investigation [11]. However, many of these approaches could not provide broad and durable protection from influenza virus drift and shift variants.

All these challenges indicate the need for more effective vaccination strategies aimed at inducing broad cross-protection not only against seasonal influenza variants but also zoonotic and emerging pandemic influenza strains. A number of conserved protein targets to elicit such cross-protective immunity have been under investigation for almost two decades, with long alpha-helix (LAH) from HA stalk and ectodomain of matrix protein 2 ion channel (M2e) being the most studied ones [20,21,22,23,24,25,26,27,28,29]. Due to the close packing to the viral envelope, these conserved antigens are immunosubdominant and natural influenza infections and current vaccination regimens elicit little or no antibody responses [20,25,30]. Yet, various strategies to elicit more potent immune responses have been developed. Antigen presentation on the surface of virus-like particles (VLPs) stands out as one of the most efficient techniques to enhance the exposure of such weak immunogens to the host immune system by displaying the antigens of choice in a symmetric, highly organized structure with a large number of repetitions [31].

However, despite the highly conserved nature, vaccines based solely on one conserved influenza antigen most often cannot provide complete protection against highly divergent heterosubtypic virus infections [21,22,23,24,25]. Furthermore, although M2e vaccine prototypes have shown promising effects in animal models, the results do not always translate well to human studies, with high-dose associated adverse reactions, rapidly decreasing antibody titers or narrow cell-mediated immunity [25]. Vaccine candidates including several conserved epitopes might confer broader and longer-lasting protection against influenza virus infections. In the present study, we investigated the immunogenicity and efficacy of a panel of broadly protective influenza vaccine prototypes based on influenza HA stalk and M2e antigens integrated into bacteriophage AP205 coat protein (CP) VLPs expressed in *Escherichia coli* system. As the termini of AP205 CP are surface exposed, it is particularly tolerant to N- and C-terminal fusions. In addition, due to the interdimer disulfide bonds, AP205 CP VLPs are very stable, making them a particularly suitable platform for carrying foreign antigens [32]. The merging of genetic fusion and chemical coupling techniques to expose two different foreign influenza antigens on a single particle without compromising the trimeric conformation of the stalk protein is a perspective approach for a broadly-effective vaccine candidate that could protect against the constantly emerging influenza virus strains. Similar VLP vaccine candidates have employed a linear LAH epitope [24,29,33]; however, such strategy does not embrace the immunological advantages of conformational epitopes [34].

## 2. Materials and Methods

### 2.1. Antigen Expression and Purification

HA tri-stalk: Production and purification of soluble H1N1 A/Luxembourg/43/2009 subtype HA tri-stalk protein were accomplished using previously reported methods [35].

3M2e protein: The gene of a 3M2e protein, corresponding to a triplet 24 aa sequence of the M2e, derived from H1N1, H5N1 and H11N9 subtypes [29] (Table S1), was synthesized and supplied by BioCat GmbH (Heidelberg, Germany), cloned in a pET24a(+) vector. The construct was originally designed for chemical coupling purposes and contained a 6His-Tag sequence along with the TEV protease cleavage site at the N-terminus and a cysteine separated by a rigid EAAAK linker at the C-terminus. The construct was expressed in *E. coli* BL21 (DE3) cells according to the manufacturer’s recommendations. For purification, cells were disrupted in lysis buffer (20 mM Tris-HCl (pH 8.0) and 300 mM NaCl) by sonication. Supernatant was passed through a 1 mL HisTrap^TM^ FF crude column (GE Healthcare, Uppsala, Sweden) in lysis buffer containing 10 mM imidazole. Bound protein was eluted with a linear gradient of 0.5 M imidazole in lysis buffer. For the final polishing, the protein was passed through the Superdex 200 10/300 GL column (GE Healthcare, Uppsala, Sweden) in 20 mM Tris-HCl (pH 8.0) and 200 mM NaCl.

AP205 and AP-M2e VLPs: The gene encoding wild-type AP205 CP [32] was introduced into the pETDuet-1 expression vector (Novagen, Merck KGaA, Darmstadt, Germany). The 72 aa sequence encoding the 3M2e protein was genetically fused to the C-terminus of AP205 CP; the final construct was designated as AP-M2e. AP205 and AP-M2e VLPs were produced in *E. coli* BL21 (DE3) cells. The cells were lysed by sonication in buffer A (20 mM Tris-HCl (pH 8.0) and 100 mM NaCl). To the supernatant, ammonium sulphate was added to 40% saturation following incubation for 1 h at +4 °C. The precipitate was dissolved in a minimal volume of 20 mM Tris-HCl (pH 8.0), and subjected to thermal treatment (30 min at +55 °C) following cooling down to the RT and centrifugation. For purification of AP205 VLPs, supernatant was passed through a size-exclusion Sepharose 4 FF matrix (GE Healthcare, Uppsala, Sweden) in buffer A. Selected fractions were loaded on an anion-exchange Fractogel TMAE (M) matrix (Merck KGaA, Darmstadt, Germany) in buffer A. Bound protein was eluted with a linear salt gradient of buffer B (20 mM Tris-HCl (pH 8.0) and 1 M NaCl). Ammonium sulphate was added to the selected fractions to the concentration of 1.5 M. For final purification, a hydrophobic-interaction Fractogel Propyl (S) matrix (Merck KGaA, Darmstadt, Germany), equilibrated with 50 mM NaHPO_4_ (pH 7.3) and 1.5 mM ammonium sulphate buffer, was used. Bound protein was linearly eluted with 25 mM NaHPO_4_ (pH 7.3) buffer. For purification of chimeric AP-M2e VLPs, soluble fraction was passed through a size-exclusion Sepharose 4 FF matrix in buffer A. Selected fractions were loaded on an anion-exchange Fractogel DEAE (M) matrix (Merck KGaA, Darmstadt, Germany) equilibrated with buffer A. Bound protein was eluted with a linear salt gradient of buffer B.

Purified proteins were aliquoted and stored at −20 °C. Protein purity was assessed by SDS/PAGE.

### 2.2. Chemical Coupling of HA Tri-Stalk Protein to VLPs via SATA Reagent

To introduce a sulfhydryl group into tri-stalk protein, SATA reagent was added according to the manufacturer’s protocol (Thermo Scientific, Rockford, IL, USA). Briefly, tri-stalk protein was combined with a 3.3-fold molar excess of SATA in DMSO and incubated for 30 min at room temperature (RT). Unreacted SATA was removed using Zeba™ Spin desalting column (Thermo Scientific, Rockford, lL, USA). Free sulfhydryl groups were generated by mixing the protein solution with 1/10 volume of deacetylation solution (0.5 M hydroxylamine, 25 mM EDTA in PBS, pH 7.2–7.5) and incubating for 2 h at RT. Hydroxylamine was removed by desalting. The sulfhydryl-modified tri-stalk was promptly used for chemical coupling to AP205 or AP-M2e VLPs. First, VLPs were mixed with a 10-fold excess of SMPH crosslinker (Thermo Scientific, Rockford, IL, USA) in DMSO and incubated for 30 min at RT. Residual crosslinker was removed by desalting. Subsequently, the amine-modified VLPs were combined with a 3-fold excess of sulfhydryl-modified tri-stalk protein and incubated for 30 min at RT; part of the unreacted tri-stalk protein was removed using Amicon-Ultra 4, 100K (Merck-Millipore, Cork, Ireland). Coupling efficiency was determined by SDS/PAGE. Single use aliquots of the conjugated proteins were stored at −20 °C.

### 2.3. Viruses and Recombinant Full-Length HA Proteins

Influenza viruses used in the experiments were PR8 (wild-type H1N1 A/Puerto Rico/8/1934 virus, Institute of Experimental Medicine, Saint Petersburg, Russia), Cal/09 (a mouse-adapted H1N1 A/California/7/2009 virus, Smorodintsev Research Institute of Influenza, Saint Petersburg, Russia), H3N2 (a mouse-adapted PR8-based reassortant H3N2 A/Philippines/2/1982 (X-79) virus, ISMMS, New York, USA), and rgH5N1 (a mouse-adapted PR8-based reassortant virus with HA and NA genes from H5N1 A/Viet Nam/1203/2004 virus, CDC, Atlanta, GA, USA). For ICS analyses, H1N1 A/California/7/2009 virus was purified by ultracentrifugation in a sucrose gradient. The viruses were propagated in eggs for 2 days at +37 °C and stored in single-use aliquots at −70 °C. All experiments with viruses were performed in a BSL2 conditions.

Recombinant full-length HA proteins used in this study, kindly provided by Professor F. Krammer (ISMMS, New York, USA), are listed as follows: cH6/1 (head domain from H6N1 A/mallard/Sweden/81/2002 virus and stalk domain from Cal/09 virus), H1 (H1N1 A/Solomon islands/03/2006), H2 (H2N2 A/Japan/305/1957), H3 (H3N2 A/Wyoming/3/2003), H4 (H4N6 A/red knot/Delaware/541/1988), H5 (H5N1 A/Indonesia/5/2005), H6 (H6N4 A/mallard/Sweden/81/2002), H7 (H7N9 A/Shanghai/1/2013), H8 (H8N4 A/mallard/Sweden/24/2002), H9 (H9N2 A/guinea fowl/Hong Kong/WF10/1999), H10 (H10N8 A/Jiangxi-Donghu/346/2013), and H11 (H11N9 A/shoveler/Netherlands/18/1999). All proteins were stored at −70 °C.

### 2.4. Animals

Eight- to ten-week-old female BALB/c and C57BL/6J mice were purchased from the laboratory breeding nursery of the Russian Academy of Sciences “Stolbovaya” (Moscow region, Russia). Mice were anesthetized for all intranasal procedures, retro-orbital bleeding and retro-orbital injections with isoflurane. The handling of animals was performed in accordance with the “Manual for laboratory animals and alternative models in biomedical research” (2010). The study design was approved by the Local Institutional Ethical Committee (ethical approval number 1/19 dated 08 February 2019).

### 2.5. Vaccination and Challenge

**Figure 1:**
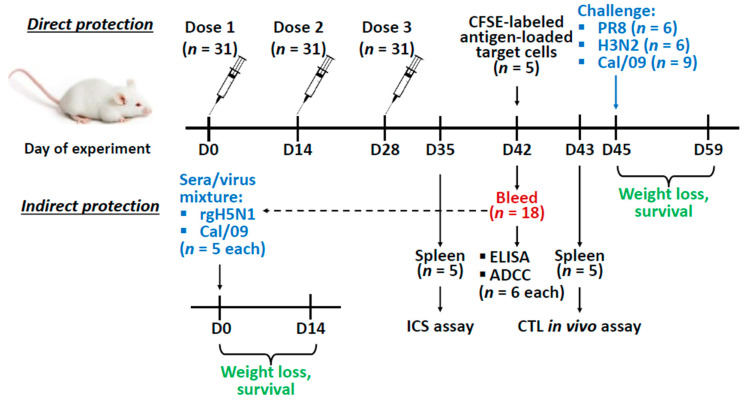
Overview of the mice study design. Mice were immunized with the recombinant vaccine prototypes three times with a two-week interval. Two weeks after the last immunization mice were bled to assess antibody titers and antibody-dependent cellular cytotoxicity (ADCC) (*n* = 6 for each), and to test the indirect protection of immune sera. For the in vivo indirect protection experiment, naïve mice were inoculated with 1:1 sera/virus mixture (3 × LD_50_ of rgH5N1 or Cal/09 (*n* = 5 for each)) and monitored daily for weight loss and survival for 14 days. In addition, on day 35 and 43 spleens (*n* = 5) were collected for the intracellular cytokine staining (ICS) assay or cytotoxic T-lymphocyte (CTL) in vivo assay respectively. On day 45 immunized mice were infected with a lethal dose of challenge virus (3 × LD_50_ of either PR8 or H3N2 (*n* = 6 for each), or 30 × LD_50_ Cal/09 virus (*n* = 9)) and monitored daily for weight loss and survival for 14 days.

Direct protection: BALB/c mice were immunized intraperitoneally with three doses of each vaccine antigen at a dose of 25 µg of protein per mouse, two weeks apart (the number of animal is indicated in Figure 1). The proteins were diluted to 0.25 mg/mL in PBS and 100 µL of antigen were combined with 100 µL of Alum adjuvant (AlumVax Hydroxide 2% ready-to-use suspension, OZ Biosciences, Marseille, France). The control group received sterile PBS with adjuvant. Two weeks after the last immunization mice were bled via retro-orbital sinus to obtain serum samples. At day 45, mice were challenged intranasally with 50 µL of 3 × LD_50_ of either PR8 or H3N2 virus, or 30 × LD_50_ of Cal/09 virus. Weight loss and survival of the challenged mice were monitored daily for 14 days post-infection. Survival was defined as a 25% weight loss for PR8 and H3N2 viruses and 30% for the Cal/09 virus.

Passive transfer: An in vivo indirect protection assay was carried out as described previously [36]. Briefly, sera from immunized or naïve mice obtained during the direct protection experiment were mixed with PBS at a 1:1 ratio and heat-inactivated at 56 °С for 1 h. Then the sera were mixed 1:1 with rgH5N1 or Cal/09 virus, to reach the dose 3 × LD_50_ and incubated at RT for 30 min. Naïve BALB/c mice were infected intranasally with a mixture of virus and sera at a volume of 50 µL and monitored for their survival rates and weight loss for 14 days post-infection (Figure 1). Survival was defined as a 25% weight loss for the rgH5N1 virus and 30% for the Cal/09 virus.

### 2.6. Enzyme Linked Immunosorbent Assay (ELISA)

For ELISA, EIA/RIA 96-Well microtitration plates (Corning Life Sciences, Tewksbury, MA, USA) were coated with 50 μL of the recombinant proteins at a concentration of 2 µg/mL overnight at +4 °C. Plates were washed with 0.05% Tween 20 in PBS (PBST) and blocked with 50 μL of 1% bovine serum albumin (BSA) in PBS for 30 min at +37 °C. Three-fold dilutions of sera were prepared starting from 1:100 (for IgG and IgG1) or 1:10 (for IgG2a) and added to the coated wells (50 µL/well). The plates were incubated for 1 h at +37 °C and washed four times with PBST. Bound IgG, IgG1, and IgG2a antibodies were detected with 50 μL of horseradish peroxidase (HRPO) conjugated goat anti-mouse IgG (Sigma-Aldrich, Saint Louis, MO, USA) or primary rabbit anti-mouse IgG1 and IgG2a antibodies (Abcam, Cambridge, UK) respectively, followed by the addition of secondary HRPO conjugated goat anti-rabbit IgG (Sigma-Aldrich, Saint Louis, MO, USA) for IgG1 and IgG2a antibody detection. Plates were incubated with primary antibody or the conjugates for 30 min at +37 °C and washed four times with PBST. The detection of antibody binding was performed with 3,3’, 5,5’;-tetramethylbenzidine substrate (1-Step Ultra TMB–ELISA Substrate Solution, Thermo Scientific, Rockford, IL, USA). The endpoint serum IgG antibody titers were determined as the last serum dilution with OD_450_ value exceeding at least twice the mean OD values of the control wells (all the components except mouse sera). For negative specimens, the titers were assigned a value of 1:50 (for IgG and IgG1) or 1:5 (for IgG2a) for group comparison purposes. The log10-transformed antibody titers were used for statistical analyses.

### 2.7. Antibody-Dependent Cellular Cytotoxicity (ADCC) Assay

The functional activity of the induced antibody was assessed by ADCC assay, performed by measuring the levels of NK-cell degranulation activity. MDCK cells were used as target cells expressing viral antigens since it is known that HA and M2 proteins are expressed abundantly on the surface of influenza virus infected cells [37]. MDCK cell monolayers, seeded in 96-well plates (tissue culture plate 96-well flat, Sarstedt, Nümbrecht, Germany) the day before, were infected with Cal/09 virus at a multiplicity of infection (MOI) 3. Two h after inoculation, conditional medium was removed and 15 µL of serum samples were added in duplicates to the wells, followed by incubation at +37 °C in a 5% CO_2_ atmosphere for 15 min. Then, 135 µL of CR-0 (2.5 mL 1 M Hepes, 5 mL Glutamax (Gibco), 5 mL antibiotic-antimycotic (Gibco), 17 µL β-mercaptoethanol, RPMI-1640 (Gibco) up to 500 mL) with 1 × 10^6^ murine splenocytes collected from naïve C57BL/6J mice were added to each sample and incubated overnight. After 16–18 h, supernatants were collected from the plate and stained with ZombieAqua fixable viability dye, anti-CD3, anti-CD49b, anti-CD45.2, anti-CD107a antibody- conjugates for 20 min in a cool dark place (Table 1). Samples were then washed twice with 200 µL of PBS. Plates were stored in a cool dark place prior to flow cytometric analysis. At least 100,000 events were measured using a Navios flow cytometer (Beckman Coulter, Brea, CA, USA). Data were analysed using FlowJo software (TriStar Inc., El Segundo, CA, USA). Gating strategy is shown in Supplementary (Figure S1).

### 2.8. Intracellular Cytokine Staining (ICS)

T-cell-mediated immune responses were analyzed by ICS to gamma-interferon (IFNγ) and tumor necrosis factor alpha (TNFα). Murine splenocytes were isolated one week after the third immunization and red blood cells were lysed by ammonium-chloride potassium lysis buffer (ACK Lysing Buffer, Thermo Scientific, Rockford, IL, USA). For in vitro stimulation, 1 × 10^6^ cells were incubated with either purified antigens (tri-stalk and 3M2e) at 1 µg/well or sucrose-gradient purified Cal/09 virus at an MOI of 1.0 EID_50_/cell for one h in 100 µL of CR-0, in 96-well microtitration U-bottom well plates (Sarstedt, Nümbrecht, Germany). Then, 50 µL of CR-30 (CR-0 with 30% FBS) was added, to the final FBS concentration of 10%. After 16–18 h, 50 µL of GolgiPlug solution (Becton Dickinson, Franklin Lakes, NJ, USA)—alone or with PMA (Phorbol myristate acetate as positive control)—were added at 1:250 dilution and the mixture was incubated for another five h. Samples were stained with ZombieAqua fixable viability dye, anti-CD4, anti-CD8, anti-CD44, anti-CD62L antibody-conjugates for 20 min in a cool dark place (Table 2).

Samples were then washed twice with 200 µL of staining buffer (0.8 g BSA, 4 mL 5% NaN_3_, PBS up to 400 mL). ICS was performed with Cytofix/Cytoperm kit (Becton Dickinson, Franklin Lakes, NJ, USA) according to the manufacturer’s instructions followed by staining samples with anti-IFNγ, and anti-TNFα antibody-conjugates for 20 min in a cool dark place (Table 3).

Then, samples were washed twice with 200 µL of wash buffer (Cytofix/Cytoperm kit). Samples were fixed in 1% paraformaldehyde and stored in a cool dark place prior to flow cytometric analysis. At least 100,000 events were measured using a Navios flow cytometer (Beckman Coulter, Brea, CA, USA). Data were analyzed using FlowJo software (TriStar Inc., El Segundo, CA, USA). Gating strategy is shown in Supplementary (Figure S2). The percentage of virus/antigen-specific T-cells was calculated by subtracting the negative control from the cytokine-positive T-cells.

### 2.9. The Cytotoxic T-Lymphocyte (CTL) In Vivo Assay

An in vivo cytotoxicity assay was performed as described by Durward et al. [38] with modifications. Briefly, target cells were prepared from splenocytes of naïve BALB/c mice (1 × 10^8^ splenocytes in 10 mL complete DMEM supplemented with 10% FBS) and loaded with either 200 µg of protein (tri-stalk or 3M2e) or an equal volume of PBS for one h at +37 °C, with occasional mixing. Next, cells were washed with PBS and stained with different concentrations of carboxyfluorescein succinimidyl ester (CFSE) in PBS (60, 15 or 3.75 mM CFSE for 3M2e, tri-stalk and PBS control, respectively). Cells were then washed, resuspended in Hanks solution, and mixed in equal amounts to the final concentration of 1 × 10^8^ cells/mL. Ten million prefiltered target cells were administered in 100 µL to anaesthetized BALB/c mice by retro-orbital injection. After 16–18 h, mice were sacrificed; splenocytes were harvested and assessed by flow cytometry. Gating strategy is shown in Supplementary (Figure S3). The ratio of peptide-loaded to control target cells was calculated as a normalized measure of protein-specific cytotoxicity between immunization groups. The ratio in placebo group samples (PBS) was assumed as survival at basal protein-specific cytotoxicity.

### 2.10. Statistical Analyses

Data were analyzed with the GraphPad Prism 6.0 software (GraphPad Software Inc., La Jolla, CA, USA). Statistical significance of immunogenicity outcomes (the log10-transformed antibody titers, ADCC levels, virus/peptide-specific T-cell levels, or the in vivo killing activity of CTLs) and protection outcomes (AUC of weight loss values) were determined by one-way ANOVA followed by a Tukey’s multiple comparison test (for comparing ELISA endpoint titer values). Differences in the survival rates after challenge were analyzed by a log-rank Mantel–Cox test.

## 3. Results

### 3.1. Antigen Design

**Figure 2:**
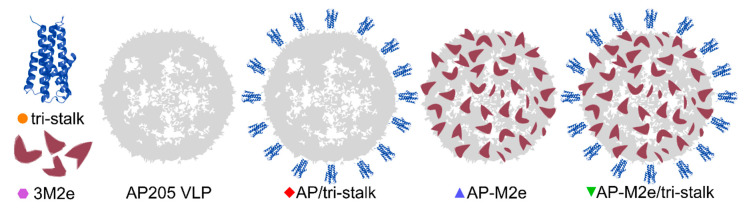
Schematics of the recombinant protein design. We produced three soluble antigens: an N-terminally extended long alpha-helix of H1 hemagglutinin (tri-stalk), a triple M2e peptide (3M2e), and a wild-type bacteriophage AP205 coat protein that self assembles into virus-like particles (VLPs). The genes of 3M2e and AP205 coat protein (CP) were genetically fused and expressed as a single protein, yielding in stable chimeric particles (AP-M2e), and the tri-stalk protein was coupled to the surface of AP205 VLPs (AP/tri-stalk) or AP-M2e VLPs (AP-M2e/tri-stalk).

In this study, to generate and test different broadly protective influenza vaccine candidates, HA tri-stalk and a triple M2e protein (3M2e) were integrated into an AP205 VLP platform, alone or in combination, creating five different broadly protective influenza vaccine prototypes (Figure 2).

**Figure 3:**
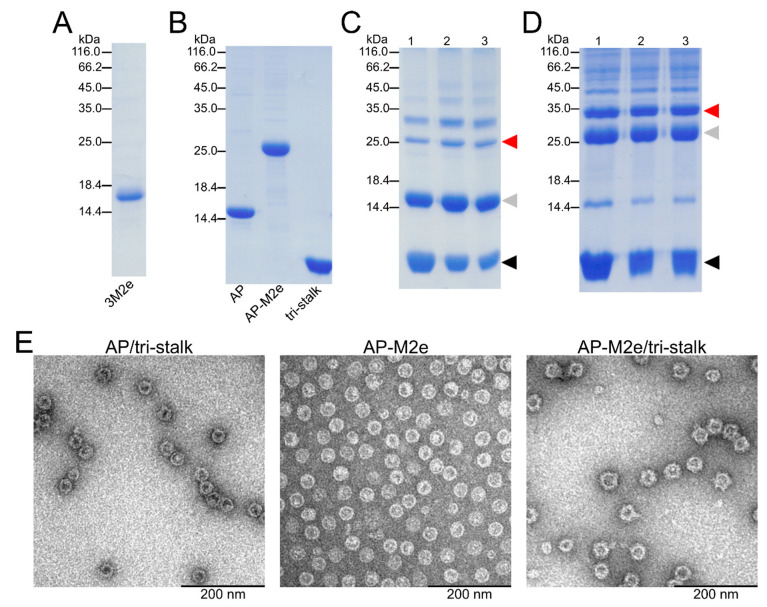
Characterization of recombinant protein constructs produced in *E. coli*. The purity of recombinant proteins—(**A**) 3M2e, (**B**) AP205, AP-M2e, and tri-stalk—was analyzed in SDS/PAGE under reducing conditions. Hemagglutinin (HA) tri-stalk protein was then coupled to the surface of (**C**) AP205 VLPs and (**D**) AP-M2e VLPs and subsequently analysed in SDS/PAGE under reducing conditions: lane 1—immediately after coupling; lane 2—after Amicon Ultra 4 filtration; lane 3—after freeze-thawing. Red arrow refers to the coupling zone, grey arrow—coat protein monomer, black arrow—tri-stalk protein. Coupling bands indicate a successful reaction. (**E**) Electron microscopy images of chimeric particles after freeze-thawing.

Since aa 10–24 of M2e peptide vary between influenza virus subtypes, combining the M2e from different subtypes can increase the vaccine-induced cross-protection [25]. Therefore, a 3M2e protein, corresponding to three variants of the conserved M2e peptide, was expressed in *E. coli* and purified until near homogeneity (Figure 3A, Table S1). The 3M2e sequence was derived from the M2e of the highly pathogenic H5N1 virus, as well as H1N1 and H11N9 subtypes to cover a wider range of influenza viruses. Noteworthy, two of the M2e fragments comprising the 3M2e protein share high sequence similarity with the M2e from H3N2 viruses. In parallel, sequence of the 3M2e was genetically fused to the C-terminus of AP205 CP yielding in expression of stable chimeric AP-M2e particles. Phage AP205 VLPs and AP-M2e VLPs were expressed in *E. coli* and purified as described in Materials and Methods section (Figure 3B).

An N-terminally extended LAH of H1 HA stalk (referred to as tri-stalk protein) was expressed and purified using a three-step chromatography method as described in our previous study (Figure 3B) [35]. Produced in *E. coli*, it forms an α-helical trimer highly similar to the corresponding region of native HA in its post-fusion form. It has been shown previously that the monomeric LAH is highly immunogenic in mice when incorporated into hepatitis B virus core particles [29,33]. Moreover, post-fusion LAH-specific antibodies have been shown to elicit a durable cross-protective immunity independent of virus neutralization activity [34]. HA tri-stalk protein was chemically coupled to the surface of purified AP205 or AP-M2e VLPs (Figure 3C,D). This was done by chemical crosslinking via the SMPH linker between the VLPs and SATA-modified tri-stalk protein. Due to the crosslinking of subunits, derivatization of VLPs shows the typical VLP ladder with monomeric and multimeric subunits. Although some aggregation was observed, the chimeric particles were stable upon freezing at −20 °C and subsequent thawing as confirmed by SDS/PAGE and electron microscopy (Figure 3E).

### 3.2. Immunization with Chimeric VLPs Generates Broadly Cross-Reactive Antibodies to Heterosubtypic Influenza A Hemagglutinins

Female BALB/c mice were intraperitoneally immunized with one of the four protein antigens (tri-stalk, AP/tri-stalk, AP-M2e, AP-M2e/tri-stalk) combined with Alum adjuvant in a prime-boost schedule with two-week intervals between the three immunizations; the control group received PBS with Alum adjuvant. Two weeks after the last immunization mice (*n* = 6) were bled and sera were analyzed for their reactivity to different influenza antigens by ELISA.

**Figure 4:**
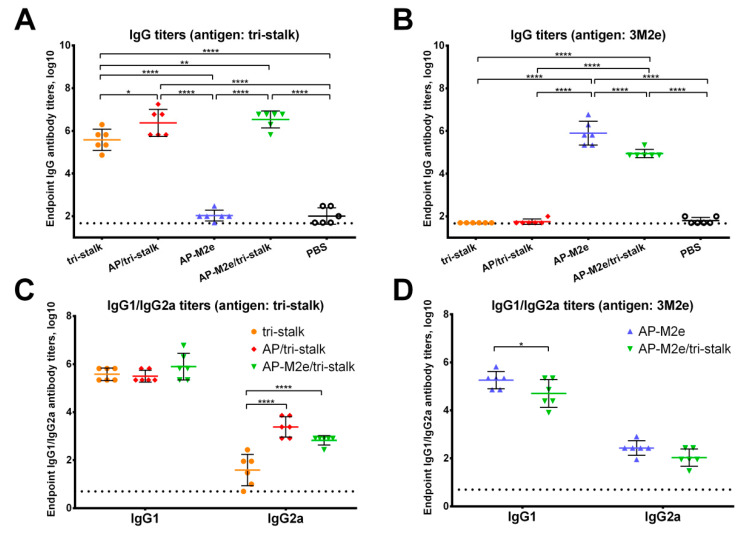
Immunogenicity of the recombinant vaccine candidates included in the study. Serum IgG antibodies from BALB/c mice (*n* = 6) immunized with vaccine candidates were tested by ELISA for their binding activity against the proteins included in the vaccine candidates: (**A**) IgG antibody binding to the tri-stalk protein; (**B**) IgG antibody binding to the 3M2e protein; (**C**) IgG1/IgG2a subclasses binding to the tri-stalk protein; (**D**) IgG1/IgG2a subclasses binding to the 3M2e protein. The dotted line indicates the limit of antibody detection; error bars represent mean ± SD. Statistical analysis was performed using one-way ANOVA (**A**–**B**) or two-way ANOVA (**C**–**D**) followed by a Tukey’s multiple comparison test (* *p* < 0.05; ** *p* < 0.01; *** *p* < 0.001; **** *p* < 0.0001).

All vaccine prototypes induced high levels of homologous serum IgG antibody against the tri-stalk and 3M2e antigens (Figure 4A,B). Significant differences in the amount of tri-stalk binding antibody were observed, with chimeric VLPs exposing tri-stalk inducing higher antibody levels than the tri-stalk protein alone. Both VLP vaccines induced comparable levels of tri-stalk targeted antibody since both constructs carry identical viral antigen on the same delivery vehicle. However, coupling of the tri-stalk antigen to the AP-M2e VLPs slightly reduced the reactivity of antisera with anti-M2e antibodies. Furthermore, we examined the IgG1/IgG2a subclass profile of the induced serum antibody. The tri-stalk antigen predominantly elicited homologous antibody of the IgG1 subclass, suggesting the induction of Th2-biased immune responses (Figure 4C). Yet, coupling of the tri-stalk to either AP205 VLPs or AP-M2e VLPs significantly increased the proportion of the tri-stalk-reactive IgG2a antibody. Since the IgG1 levels between these three vaccine groups were comparable, the differences in the total IgG antibody were driven mainly by the IgG2a subclass. Similarly, although the majority of M2e-binding antibodies were of IgG1 subclass, some levels of M2e-reactive IgG2a antibody were also detected (Figure 4D). For the 3M2e antigen, the differences in the immunogenicity between the two M2e-containing vaccines were driven by the IgG1 subclass antibody, further suggesting that the induction of IgG2a antibody is dependent on the VLP vehicle, rather than the antigen itself.

**Figure 5:**
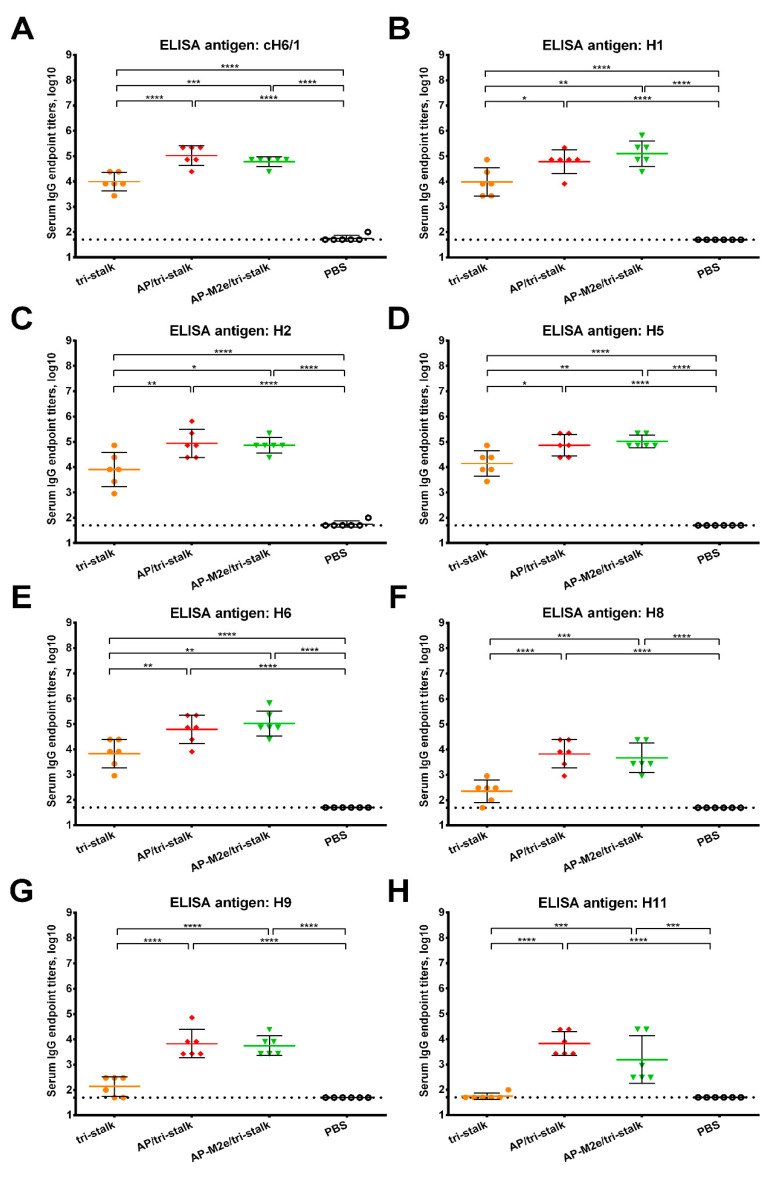
Cross-reactivity of the recombinant vaccine candidates against group 1 hemagglutinins. Breadth of the antibody response elicited by the immunization of BALB/c mice (*n* = 6) with the vaccine prototypes was determined by ELISA against a panel of full-length recombinant HA proteins: (**A**) cH6/1—head domain from H6N1 A/mallard/Sweden/81/2002 virus and stalk domain from H1N1 A/California/04/2009 virus; (**B**) H1N1 A/Solomon islands/03/2006; (**C**) H2N2 A/Japan/305/1957; (**D**) H5N1 A/Indonesia/5/2005; (**E**) H6N4 A/mallard/Sweden/81/2002; (**F**) H8N4 A/mallard/Sweden/24/2002; (**G**) H9N2 A/guinea fowl/Hong Kong/WF10/1999; (**H**) H11N9 A/shoveler/Netherlands/18/1999. The dotted line indicates the limit of antibody detection; error bars represent mean ± SD. Statistical analysis was performed using one-way ANOVA followed by a Tukey’s multiple comparison test (* *p* < 0.05; ** *p* < 0.01; *** *p* < 0.001; **** *p* < 0.0001).

**Figure 6:**
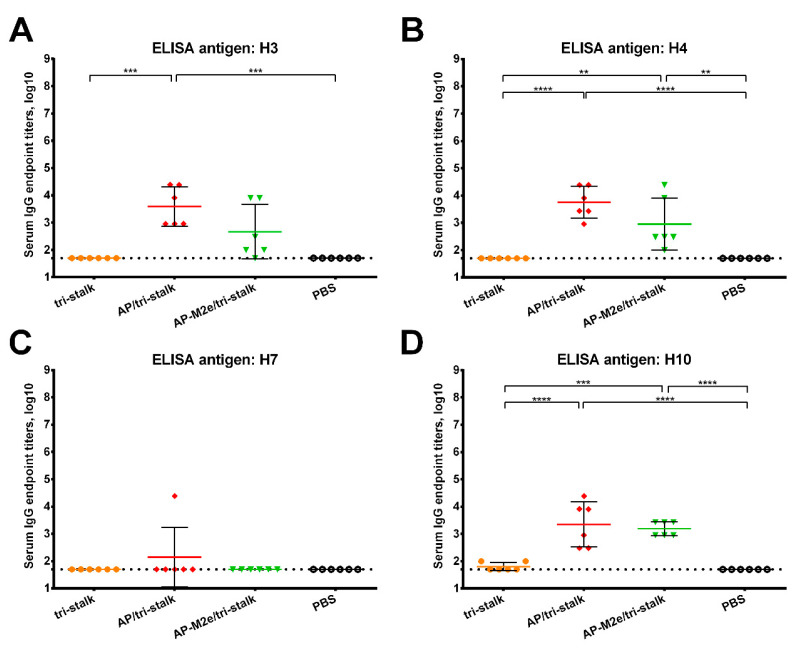
Cross-reactivity of the recombinant vaccine candidates against group 2 hemagglutinins. Breadth of the antibody response elicited by the immunization of BALB/c mice (*n* = 6) with the vaccine prototypes was determined by ELISA against a panel of full-length recombinant HA proteins: (**A**) H3N2 A/Wyoming/3/2003; (**B**) H4N6 A/red knot/Delaware/541/1988; (**C**) H7N9 A/Shanghai/1/2013; (**D**) H10N8 A/Jiangxi-Donghu/346/2013. The dotted line indicates the limit of antibody detection; error bars represent mean ± SD. Statistical analysis was performed using one-way ANOVA followed by a Tukey’s multiple comparison test (** *p* < 0.01; *** *p* < 0.001; **** *p* < 0.0001).

Furthermore, we assessed the reactivity of the serum IgG antibody with a panel of full-length recombinant group 1 and 2 HA proteins (Figure 5 and Figure 6). As the AP-M2e vaccination group had no HA-targeted antibody, sera from this group were not included in the cross-reactivity analysis. Chimeric cH6/1 HA contained an irrelevant head domain from avian H6N1 virus and a stalk originated from Cal/09 virus, perfectly matching the sequence of the tri-stalk protein. High levels of homologous cH6/1-binding antibody were observed in the AP/tri-stalk and AP-M2e/tri-stalk vaccination groups, with lower levels identified in the group vaccinated with tri-stalk protein alone (Figure 5A). Similarly, both chimeric VLPs elicited broad antibody responses against other heterosubtypic group 1 HA proteins (pre-2009 H1, H2, H5, H6, H8, H9, and H11) whereas antisera from the tri-stalk vaccine group had weak cross-reactivity (Figure 5B–H), showing that proper presentation of the viral antigen on the VLP platform can significantly improve the cross-reactive potential of the induced antibody. In addition, AP/tri-stalk induced antisera cross-reacted substantially with group 2 H3, H4, and H10 HAs, but not against the H7 HA protein (Figure 6). Some group 2 cross-reactivity was observed in the AP-M2e/tri-stalk vaccination group as well, however, it was lower than in the AP/tri-stalk group, suggesting that the addition of the 3M2e antigen to the AP/tri-stalk vaccine could reduce cross-reactivity of HA-binding antibody.

### 3.3. Chimeric 3M2e Exposing VLPs Afford Full Protection Against Heterologous and Heterosubtypic Influenza Challenge

**Figure 7:**
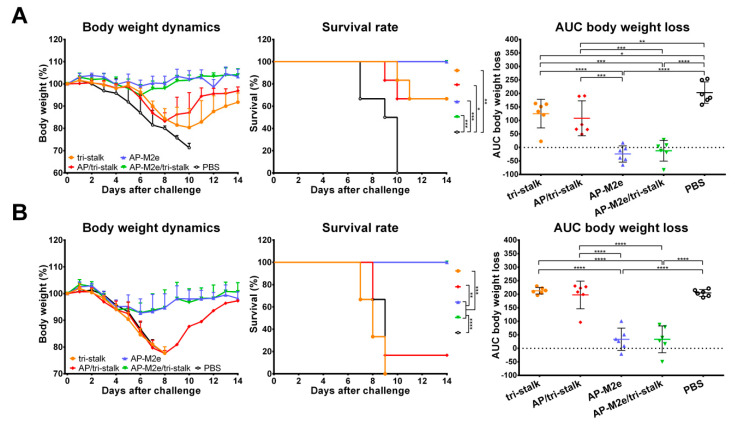
Protective immunity induced in mice immunized with recombinant vaccine candidates. BALB/c mice were vaccinated with Alum-adjuvanted recombinant proteins or placebo group samples (PBS) at days 0, 14, and 28. 17 days after the final vaccination mice were challenged with 3 × LD_50_ of (**A**) H1N1 A/Puerto Rico/8/1934 virus (*n* = 6) or with (**B**) H3N2 A/Philippines/2/1982 (X-79) virus (*n* = 6) and monitored for body weight loss (left) and survival (middle) for 14 days. Right panel shows analysis of body weight loss by measuring the area under the curve (AUC, relative to 100% value) for each mouse. For mice that died or were humanely euthanized a 75% value was assigned to all subsequent days for the purpose of statistical analysis. Error bars represent mean ± SD. The survival rates were analyzed by Mantel–Cox test, and the AUC values were compared using one-way ANOVA followed by a Tukey’s multiple comparison test (* *p* < 0.05; ** *p* < 0.01; *** *p* < 0.001; **** *p* < 0.0001).

To test the cross-protective efficacy of the recombinant protein vaccine candidates, groups of 6 BALB/c mice, immunized with the indicated immunogens, were challenged with lethal doses of heterologous and heterosubtypic influenza viruses (Figure 7). The antibody responses induced by AP-M2e and AP-M2e/tri-stalk immunizations fully protected mice against a lethal unmatched PR8 virus challenge (Figure 7A). Mice from these two groups displayed minimal signs of disease, measured as weight loss, and completely recovered from the infection. Tri-stalk protein and AP/tri-stalk VLPs provided partial 66.7% protection against PR8 virus challenge, although these vaccination groups experienced significant weight loss. Furthermore, while HA stalk-based vaccine prototypes often elicit incomplete cross-group protection, limited to the sequence similarity between the immunization antigen and challenge virus [21,22,23,24], all AP-M2e and AP-M2e/tri-stalk vaccinated animals were completely protected from the group 2 H3N2 virus challenge. The weight loss during the challenge phase did not exceed 10% (Figure 7B). In contrast to the PR8 challenge, immunization with the tri-stalk protein and AP/tri-stalk vaccine candidates did not protect mice against death and weight loss caused by the heterosubtypic H3N2 virus, suggesting that the M2e-targeted antibody were the main contributors to the cross-protection. 

### 3.4. Antibodies Elicited by Chimeric VLPs Protect Mice Against rgH5N1 Viral Challenge in a Passive Serum Transfer Challenge Experiment

**Figure 8:**
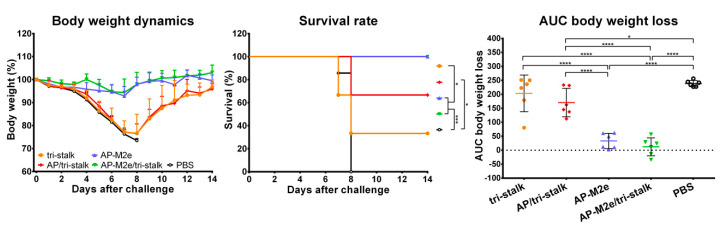
Indirect protection of immune sera. Naïve BALB/c mice (*n* = 6) were inoculated with a mixture of immune sera and 3 × LD_50_ rgH5N1 A/Viet Nam/1203/2004 virus and monitored for average weight changes (left) and survival (middle) for 14 days post-challenge. Right panel shows analysis of body weight loss by measuring the area under the curve (AUC, relative to 100% value) for each mouse. For mice that died or were humanely euthanized a 75% value was assigned to all subsequent days for the purpose of statistical analysis. Error bars represent mean ± SD. The survival rates were analyzed by Mantel–Cox test, and the AUC values were compared using one-way ANOVA followed by a Tukey’s multiple comparison test (* *p* < 0.05; *** *p* < 0.001; **** *p* < 0.0001).

We also performed an in vivo passive transfer experiment. Immune sera, collected as described above, was mixed with 3 × LD_50_ of rgH5N1 challenge virus and intranasally administered to groups of 6 naïve BALB/c mice. Sera from AP-M2e and AP-M2e/tri-stalk immunized mice possessed a strong cross-protective activity against heterosubtypic lethal rgH5N1 virus showing a 100% survival rate and no significant weight loss (Figure 8). AP/tri-stalk immune sera conferred partial protection against the rgH5N1 virus challenge, and mice from this group exhibited significantly decreased weight loss as compared to the negative control group. In contrast, sera from the tri-stalk immunized mice were not cross-protective, suggesting that the VLP delivery of the tri-stalk protein can significantly improve the breadth of antibody reactivity. Similar to the direct protection experiment, these data demonstrate that the M2e-based vaccines provide full protection against the rgH5N1 challenge, whereas the tri-stalk antigen can only partially protect mice against this virus.

### 3.5. Chimeric AP-M2e/Tri-Stalk VLPs Confer Robust Protection Against a High Dose of Homologous H1N1 A/California/7/2009 Influenza Challenge

We conducted an additional challenge experiment with a high dose of homologous Cal/09 virus. For the direct protection study, groups of 9 BALB/c mice were vaccinated as described above, with an additional vaccination group immunized with an Alum-adjuvanted 3M2e protein. All vaccine constructs were very immunogenic, as showed by ELISA, with serum IgG antibody raised to the corresponding antigen present in each vaccine candidate, affirming the data obtained in at the previous experiments and confirming that the immunizations were successful (Figure S4).

**Figure 9:**
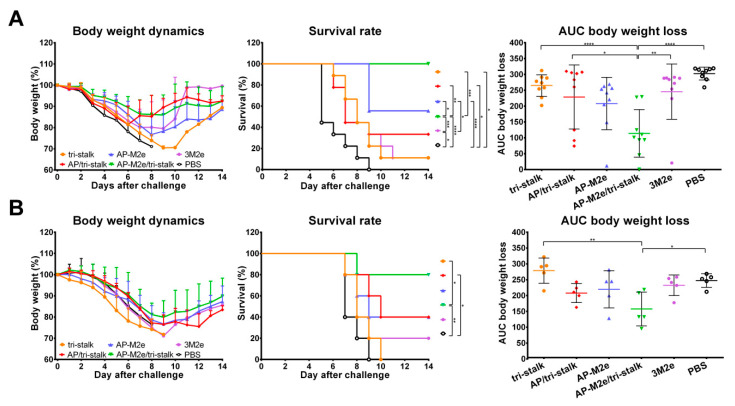
Immune protection conferred against lethal H1N1 A/California/7/2009 influenza virus challenge. (**A**) BALB/c mice were vaccinated with Alum-adjuvanted recombinant proteins or PBS at days 0, 14, and 28. 17 days after the final vaccination mice (*n* = 9) were challenged with 30 × LD_50_ of Cal/09 virus and monitored for 14 days post-challenge. (**B**) Naïve BALB/c mice (*n* = 5) were inoculated with a mixture of immune sera and 3 × LD_50_ Cal/09 virus and monitored for 14 days post-challenge. Left panel shows dynamics of body weight change; middle panel shows survival rates; right panel shows analysis of average body weight loss by measuring the area under the curve (AUC, relative to 100% value) for each mouse. For mice that died or were humanely euthanized a 75% value was assigned to all subsequent days for the purpose of statistical analysis. Error bars represent mean ± SD. The survival rates were analyzed by Mantel–Cox test, and the AUC values were compared using one-way ANOVA followed by a Tukey’s multiple comparison test (* *p* < 0.05; ** *p* < 0.01; *** *p* < 0.001; **** *p* < 0.0001).

Vaccinated mice were challenged with 30 × LD_50_ of the Cal/09 virus. Antibody responses induced by all five vaccine candidates delayed the infection and significantly improved the survival rates of mice after infection (Figure 9A). However, the tri-stalk protein, AP/tri-stalk, AP-M2e, and 3M2e vaccine prototypes did not protect animals from a significant weight loss, and only 11.1–55.6% of mice survived the infection. In contrast, an impressive 100% survival was achieved in the AP-M2e/tri-stalk group, with no significant weight loss observed. Noteworthy, the AP-M2e/tri-stalk vaccine afforded significantly better protection against this challenge than the AP-M2e vaccine.

For the indirect protection study, immune sera from the vaccinated mice were combined with 3 × LD_50_ of the Cal/09 virus and groups of 5 BALB/c mice were inoculated as described above. Only the sera from AP/tri-stalk and AP-M2e/tri-stalk immunized mice possessed a strong protective activity against the homologous Cal/09 virus (Figure 9B). In addition, the AP-M2e/tri-stalk immune sera significantly enhanced survival rates as compared to the tri-stalk and AP/tri-stalk groups, and it was the only vaccine candidate to protect animals against significant weight loss. These data suggest that the tri-stalk targeted antibodies are critical for the protection against a homologous influenza virus infection, and the antibodies targeted to M2e play a supporting role.

### 3.6. Protection Induced by Recombinant Vaccine Prototypes is Mediated by Fc-Receptor Dependent Effector Mechanisms

**Figure 10:**
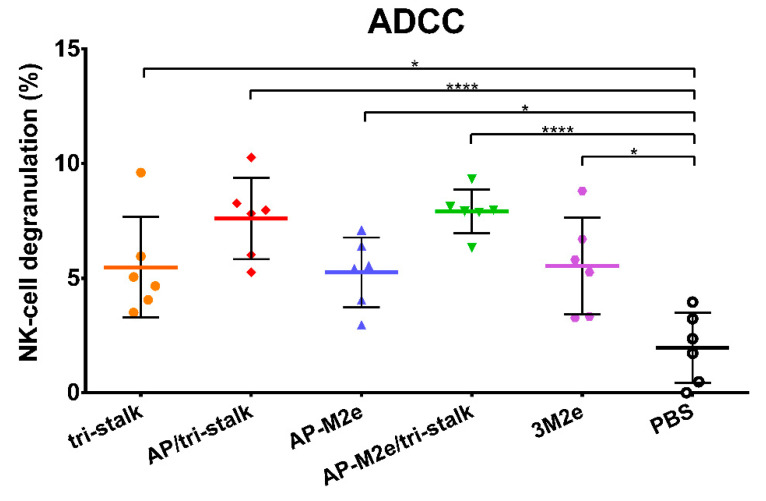
Antibody-dependent cellular cytotoxicity (ADCC) of the immune sera. Serum samples of BALB/c mice (*n* = 6) immunized with the recombinant vaccine candidates were assessed in duplicates for their ability to induce NK-cell degranulation measured by flow cytometry. Error bars represent mean ± SD. The NK-cell degranulation values were compared using one-way ANOVA followed by a Tukey’s multiple comparison test (* *p* < 0.05; **** *p* < 0.0001).

Levels of ADCC were measured by determining degranulation activity of mouse NK-cells under the influence of the immune sera. NK-cells recognize viral antigens on the surfaces of virus-infected cells and directly induce their clearance [39]. MDCK cells expressing Cal/09 viral proteins were used as target cells capturing virus-specific antibodies from immune sera. The NK-cell degranulation induced by the Fc-domains of the captured antibody was assessed by flow cytometric analysis. All five vaccine prototypes induced significant degranulation by NK-cells (Figure 10), however, more distinct ADCC activity was seen for the AP/tri-stalk and AP-M2e/tri-stalk immune sera. Although the difference between the AP-M2e and AP-M2e/tri-stalk groups was not significant by the ANOVA test, a direct comparison of these groups using a non-parametric Mann–Whitney U test revealed a strong significance (*p* = 0.0087), suggesting that the tri-stalk targeted antibodies possess stronger ADCC activity than the M2e-targeted antibody.

### 3.7. M2e Containing Vaccines Induce T-Cell Mediated Immune Responses

**Figure 11:**
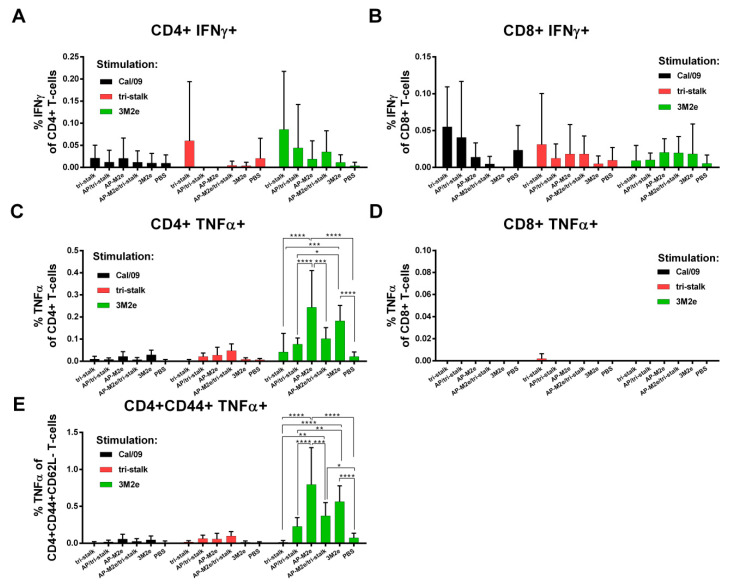
T-cell responses of immunized mice. BALB/c mice (*n* = 5) were immunized with recombinant vaccine candidates to measure the levels of virus/protein-specific T-cell responses by intracellular cytokine staining assay: (**A**) IFNɤ-secreting CD4^+^ T-cell responses; (**B**) TNFα-secreting CD8^+^ T-cell responses; (**C**) TNFα-secreting CD4^+^ T-cell responses; (**D**) IFNɤ-secreting CD8^+^ T-cell responses; (**E**) TNFα-secreting effector memory CD4^+^ T-cell subsets (CD44^+^CD62L^−^). Error bars represent mean ± SD. The T-cell levels were compared using two-way ANOVA followed by a Tukey’s multiple comparison test (* *p* < 0.05; ** *p* < 0.01; *** *p* < 0.001; **** *p* < 0.0001).

The levels of virus/protein-specific CD4^+^/CD8^+^ T-cells were assessed by ICS assay. A week after the last immunization, spleens were isolated from immunized BALB/c mice (*n* = 5). Splenocytes were stimulated with one of the antigens (whole Cal/09 virus, tri-stalk, 3M2e) in vitro. Neither vaccine candidate could induce significant levels of CD8^+^ T-cell responses to any of the antigens (Figure 11, right panel). There were no significant increases in the population of Cal/09 and tri-stalk specific CD4^+^ T-cell levels after immunizations (Figure 11, left panel). However, vaccination with 3M2e and AP-M2e significantly increased the population of M2e-specific TNFα-secreting CD4^+^ T-cells (Figure 11C). Interestingly, chemical attachment of the tri-stalk protein to AP-M2e VLPs led to the decrease of the M2e-specific TNFα-secreting CD4^+^ T-cells (Figure 11C). Analysis of effector memory CD4^+^ T-cell subsets (CD44^+^CD62L^−^) led to similar findings: only M2e-containing vaccines could induce significant levels of the M2e-specific CD4 T-cells (Figure 11E).

**Figure 12:**
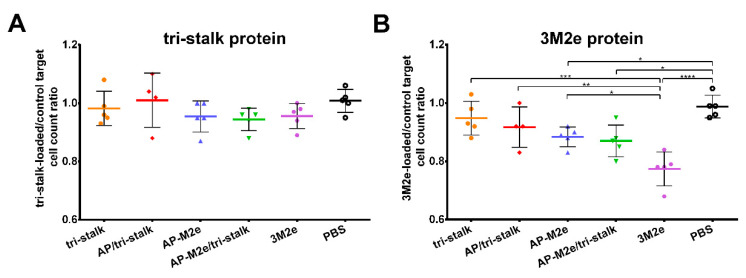
In vivo cytotoxicity of the T-cells in immunized mice. Cytotoxic activity of the induced T-cell immune responses was measured in BALB/c mice (*n* = 5) after immunization with recombinant vaccine candidates: (**A**) tri-stalk loaded target cells; (**B**) 3M2e loaded target cells. Error bars represent mean ± SD. The proportions of protein-loaded to the PBS-loaded target cells between the vaccine groups were compared using one-way ANOVA followed by a Tukey’s multiple comparison test (* *p* < 0.05; ** *p* < 0.01; *** *p* < 0.001; **** *p* < 0.0001).

The functional activity of cytotoxic T-cells was measured by a CTL in vivo assay. Splenocytes from naïve BALB/c mice were loaded with antigens (tri-stalk, 3M2e) or PBS, differentially labelled with various concentrations of CFSE. This mixture was then administered to BALB/c mice (*n* = 5), immunized as described previously. On the following day, spleens were collected to count the proportion of tri-stalk or M2e loaded cells to the control cells loaded with PBS. Surprisingly, despite the absence of M2e-specific CD8^+^ T-cells, the immune system of mice vaccinated with M2e-containing vaccine candidates could efficiently recognize and kill the M2e-loaded target cells (Figure 12B). Intriguingly, the 3M2e protein alone induced the highest level of functional cytotoxic T-cells, and the killing activity was significantly higher than in the AP-M2e and AP-M2e/tri-stalk groups. No significant cytotoxic activity against tri-stalk loaded target cells was detected in any group of immunized mice (Figure 12A).

## 4. Discussion

Evolutionarily conserved influenza antigens could potentially provide broad-spectrum protection against highly variable influenza virus subtypes, while significantly reducing the time and costs of vaccine production. Furthermore, recombinant vaccines help to avoid potentially harmful influenza viruses grown in embryonated eggs or cell cultures and therefore attract significant research interest. Here we evaluated the antigenicity and protective potential of recombinant influenza vaccine prototypes, generated by combining the methods of genetic fusion and covalent coupling to present the otherwise poor immunogens HA tri-stalk and 3M2e on the surface of AP205 VLPs.

The sequence and structure of the HA stalk are relatively conserved [20,40], highlighting HA stalk as a prospective component for a broadly protective influenza vaccine. However, with a few exceptions [41,42,43], a great part of the stalk-specific antibodies have a group-specific binding profile, dependent on the structural divergence between group 1 and group 2 HAs [44,45,46,47]. Comparison of aa sequences of N-terminally extended LAH fragment of HA proteins used in this study revealed that the observed differences in cross-reactivity of the tri-stalk induced antibody also correlate with the conservation difference between the immunization antigen and HA proteins (Figure 5 and Figure 6, Table S2). The HA stalk-targeted antibodies were cross-reactive and could efficiently recognize HA proteins from heterologous group 1 influenza viruses; notably, cross-reactivity with group 2 antigens was also observed in the AP/tri-stalk vaccination group, and to some extent—in the AP-M2e/tri-stalk vaccination group. In general, higher sequence identity was associated with increased cross-reactivity, with H7 LAH protein being the most distant from the tri stalk fragment, thus demonstrating the lowest cross-reactivity of the induced antibody. The delivery of the tri-stalk antigen using the AP205 VLP platform significantly improved the cross-reactivity of the HA-based antibody. Accordingly, the assessment of IgG antibody subclasses revealed that our vaccine candidates predominantly elicited the IgG1 antibodies, while the delivery of the virus antigens by the AP205 VLPs promoted switching to the IgG2a subclass. Mouse IgG subclasses play different roles in the antiviral immunity and their biological activities are dependent on the binding affinities to the activating and inhibitory Fc gamma receptors (FcγRs), with IgG1 subclass considered less protective than the IgG2 subclass [48,49,50]. The vehicle-based shifting to IgG2a subclass might be linked to the VLP-packed RNA as removal of bacteriophage VLP RNA has been shown to result in a shift of the induced IgG isotypes from IgG2a/c to IgG1 [51]. However, even prevailing induction of IgG1 subclass of post-fusion LAH-specific antibodies can provide significant protection against lethal heterologous virus infection [34].

Consequently, the tri-stalk antigen was capable to confer partial protection against lethal heterologous PR8 virus infection, with which the tri-stalk shares a high sequence similarity (Table S3). Consistent with previous reports [21,22,23,24], the protective immunity induced by the tri-stalk protein and AP/tri-stalk vaccines was substantially diminished when challenged with a group 2 virus. Even though the tri-stalk binding antibody had some level of cross-reactivity with the H3 HA protein, they were unable to protect mice against the H3N2 virus challenge, further indicating that the stalk-reactive antibodies afford little cross-protection between group 1 and 2 influenza viruses. Importantly, H3N2 viruses show the highest degree of antigenic drift when compared to other seasonal influenza viruses, posing the greatest risk of unmatched seasonal vaccine formulations and therefore the lowest seasonal vaccine effectiveness [7,8].

Although many researchers highlight the necessity to create a multi-component vaccine including epitopes from both group 1 and group 2 viruses to achieve a complete cross-protection against type A influenza [22,23,24], here we bypass the issue of insufficient cross-protective efficacy by incorporating both an H1 derived tri-stalk and a conserved 3M2e protein in a single recombinant immunogen. Even though our M2e-based vaccine candidates predominantly induced antibodies of IgG1 subclass, M2e-specific IgG1 antibodies have been shown to result in significant protection against divergent influenza viruses, probably because the high level of IgG1 antibodies could compensate their low affinity to activated FcγRs [52]. Likewise, in this study, the addition of the 3M2e antigen to the AP/tri-stalk antigen increased the cross-protective efficiency of the chimeric VLPs to an impressive 100% when challenged with heterosubtypic group 1 and group 2 viruses. Unlike observed in previous studies [26,27], challenged AP-M2e/tri-stalk vaccinated mice did not experience significant disease symptoms. However, it should be noted that the H3N2 virus carries internal genes from the PR8 virus (including the M2e peptide) (Table S3), therefore the M2e-targeted antibody had a comparable protective effect in both challenge groups. Similar protection results have recently been achieved by a 5xM2e VLP antigen, produced in the baculovirus expression system [28]; yet, our approach allows for a cheaper and more efficient high-yield antigen production, an important hallmark for a broadly protective influenza vaccine, accessible to the general public. Even though in this study we used the *E. coli* expression system, characterized by the presence of bacterial endotoxins, our extensive experience with yeast expression systems could easily help to overcome this problem in the future [33,53,54].

An in vivo passive transfer-challenge experiment demonstrated that the induced cross-protection is mainly antibody-mediated but acts by different modes. The tri-stalk targeted antibodies elicited by the AP/tri-stalk immunization were able to partially protect mice against lethal heterosubtypic rgH5N1 virus challenge, whereas sera from the tri-stalk immunized mice failed to protect animals. The M2e-specific antibodies were even more effective, resulting in full protection against the clinical manifestations of the infection. In this study, only the levels of IgG antibody binding were studied as a measure of immunogenicity and cross-reactivity of the vaccine, without testing their neutralizing capacity, as we did not expect high levels of virus-neutralizing antibody. Although some anti-stalk antibodies can directly neutralize the influenza virus [41,42,43], antibodies against M2e do not possess virus-neutralizing activity [25,55]. Yet, a great part of the stalk and M2e specific antibodies confer Fc-receptor dependent protection mediated by the effector mechanisms such as ADCC [20,25,48,55]. Here, the ADCC assay demonstrated that the tri-stalk induced protection is mostly dependent on the antibody effector functions, thus preserving the protective potential in serum transfer experiments. In contrast, the M2e-based protection is mediated both by the antibody Fc-receptor effector mechanisms and by some T-cell based activity, which has been shown to correlate with better vaccine-induced protection in the elderly and to confer a durable protective efficiency [56,57]. Also, it was previously demonstrated that the CD4^+^ T-cells can possess a cytolytic function on influenza-infected cells in mice, other than their classical helper role [58,59]. It remains to be investigated whether the M2e-specific CD4^+^ T-cells possess direct cytotoxic activity, or if there are other immune cells with cytotoxic potential which are induced by the M2e-based vaccine candidates. Additional challenge experiment with a high dose of homologous H1N1 influenza virus emphasized the important role of tri-stalk targeted antibody as in this experiment the tri-stalk antibodies were the main mediators of protection. Although M2e-binding antibodies were also involved in mediating the protection, a passive serum transfer experiment showed that they were not sufficient to defend mice against the infection. It is therefore not surprising that the best protective effect could be achieved when these conserved antigens were combined in single vaccine preparation.

The study has several limitations. First, the performance of the vaccine candidates was assessed only by measuring immune responses and monitoring weight loss and survival upon a lethal influenza virus challenge. Assessment of virus replication in the mouse respiratory tract could provide additional evidence for the ability of the induced antibody and T-cells to clear the virus but would require a much larger number of mice for this study. Nevertheless, for the vaccines that do not induce neutralizing antibodies the assessment of vaccine efficacy only by a clinical endpoint is widely accepted [60,61,62]. Finally, the immunogenicity of the constructed VLPs was evaluated only in the presence of an adjuvant, yet there are multiple confirmations that VLPs contribute to robust and durable IgG responses themselves due to their highly repetitive and particulate properties [63].

## 5. Conclusions

The combination of multiple conserved influenza antigens into a multivalent single-component AP-M2e/tri-stalk vaccine protected mice from high dose homologous influenza challenge and induced robust heterosubtypic immunity. Since persistent vaccine-induced immunity is a highly desirable feature of a broadly protective influenza vaccine, it should be noted that both the post-fusion LAH- and M2e-based vaccines are known to elicit long-lasting antibody responses [34,55]. Therefore, the long-term immune responses induced by the AP-M2e/tri-stalk vaccine candidate could be the subject of our future research. The vaccine candidates can be further improved by changing their formulation for intranasal delivery since intranasal administration of VLPs with relevant mucosal adjuvants has been shown to induce strong and durable cross-protective immunity [64,65]. Taken together, these data suggest that the AP-M2e/tri-stalk vaccine prototype might provide broad protection against seasonal and emergent influenza strains, thus replacing the current vaccination strategies.

## Figures and Tables

**Table 1:** A mixture of antibody-conjugates (Biolegend, San Diego, CA, USA) used for ADCC.

Mixture Component	Antibody	Dye	Cat.no	µL per Sample
1	Anti-CD3	FITC	121420	0.25
2	Anti-CD49b	PE	108907	0.25
3	Anti-CD45.2	Pacific Blue	109820	0.25
4	Anti-CD107a	APC-Cy7	104435	0.25
	or Iso anti-CD107a	APC-Cy7	400524	or 0.25
5	ZombieAqua	BV-510	123102	0.3
6	PBS	-	-	98.7

APC: allophycocyanin; PE: phycoerythrin; BV: brilliant violet; FITC: fluorescein isothiocyanate.

**Table 2:** A mixture of antibody-conjugates (Biolegend, San Diego, CA, USA) used for T-cell staining.

Mixture Component	Antibody	Dye	Cat.no	µL per Sample
**1**	Anti-CD4	PC5.5	100540	0.25
**2**	Anti-CD8	APC-Cy7	100714	0.25
**3**	Anti-CD44	PE	103008	0.25
**4**	Anti-CD62L	BV-421	104435	0.25
**5**	ZombieAqua	BV-510	123102	0.3
**6**	Staining buffer	-	-	98.7

PC5.5: PerCP/Cyanine.5; APC: allophycocyanin; PE: phycoerythrin; BV: brilliant violet.

**Table 3:** A mixture of anti-IFNγ, and anti-TNFα antibody-conjugates (Biolegend, San Diego, CA, USA).

Mixture Component	Antibody	Dye	Cat.no	µL per Sample
**1**	Anti-IFN-γ	FITC	505806	0.4
**2**	Anti-TNF-α	APC	506314	0.4
**3**	Isotype TNF-α	APC	400418	or 0.4
**4**	Wash buffer	-	-	49.2

IFNγ: interferon gamma; TNFα: tumor necrosis factor alpha; APC: allophycocyanin; FITC: fluorescein isothiocyanate.

## Supplementary Materials

The following are available online at https://www.mdpi.com/2076-393X/8/2/197/s1, Figure S1: Gating strategy for NK-cell degranulation assay (antibody-dependent cellular cytotoxicity, ADCC), Figure S2: Gating strategy for intracellular cytokine staining (ICS) assay, Figure S3: Gating strategy in flow cytometry analysis of target cells for CTL in vivo assay, Figure S4: Immunogenicity of the recombinant vaccine candidates included in the study, Table S1: Amino acid sequences of the M2e fragments comprising the 3M2e protein used in this study, Table S2: Comparison of amino acid sequences of N-terminally extended long alfa-helix fragment of various influenza A HA proteins used in this study, Table S3: Sequence conservation between the vaccination antigens and the corresponding protein fragments of influenza A challenge viruses used in this study.
