# Supplementary material for: Construction and Immunogenicity of a Novel Multivalent Vaccine Prototype Based on Conserved Influenza Virus Antigens
Source: Vaccines (Basel). 2020 Apr 24;8(2):197. doi: 10.3390/vaccines8020197 (PMC7349063; doi:10.3390/vaccines8020197)
Supplement: Supplementary file 1 [file vaccines-08-00197-s001.zip › Supplementary Tables.pdf]

Table S1. Amino acid sequences of the M2e fragments comprising the 3M2e protein used in this study.

Reference Virus	Amino Acid Sequence	1	2
Wild-type sequence of origin *	MSLLTEVETPIRNEWGCRCNGSSD		
3M2e fragment 1 **	MSLLTEVETPIRNEWGSRSNGSSD		
3M2e fragment 2	MSLLTEVETPTRNGWGSKSNGSSD		
3M2e fragment 3	MSLLTEVETPTRNEWESRSSGSSD		
**Fragment 1**	MSLLTEVETPIRNEWGSRSNGSSD		
H1N1 (A/Puerto Rico/8/1934)	MSLLTEVETPIRNEWGCRCNGSSD	2	91.7
H3N2 (A/Sofia/1250/2006)	MSLLTEVETPIRNEWGCRCNGSSD	2	91.7
**Fragment 2**	MSLLTEVETPTRNGWGSKSNGSSD		
H11N9 (A/duck/Yunnan/1282/2007)	MSLLTEVETPTRNGWGCKCSGSSD	3	87.5
**Fragment 3**	MSLLTEVETPTRNEWESRSSGSSD		
H5N1(A/white-backed munia/Hong Kong/2469/2006)	MSLLTEVETPTRNEWECRCSGSSD	2	91.7
H3N2 (A/swine/Quebec/1262080/2010)	MSLLTEVETPTRNEWECRCSGSSD	2	91.7

Note: * The most common variant found in H1N1 (e.g. A/Puerto Rico/8/1934), identical to H3N2 (e.g. A/Sofia/1250/2006); ** C changed to S to avoid the formation of SS-bonds; 1—Number of mutations within the M2e peptide; 2—% identity.

Table S2. Comparison of amino acid sequences of N-terminally extended long alfa-helix fragment of various influenza A HA proteins used in this study.

Reference Virus	Amino Acid Sequence	1	2
H1N1 A/Luxembourg/43/2009 (tri-stalk antigen)	MNTQFTAVGKEFNHLEKRIENLNKKVDDGFLDIWTYNAELLVLLENERTLDYHDSNVKNLYEKVRSQLKNNA		
H1N1 A/California/04/2009	MNTQFTAVGKEFNHLEKRIENLNKKVDDGFLDIWTYNAELLVLLENERTLDYHDSNVKNLYEKVRSQLKNNA	0	100
H1N1 A/Solomon islands/03/2006	MNTQFTAVGKEFNKLERRMENLNKKVDDGFIDIWTYNAELLVLLENERTLDFHDSNVKNLYEKVKSQLKNNA	6	91.7
H5N1 A/Indonesia/5/2005	MNTQFEAVGREFNNLERRIENLNKKMEDGFLDVWTYNAELLVLMENERTLDFHDSNVKNLYDKVRLQLRDNA	13	81.9
H2N2 A/Japan/305/1957	MNTQFEAVGKEFSNLERRLENLNKKMEDGFLDVWTYNAELLVLMENERTLDFHDSNVKNLYDKVRMQLRDNV	15	79.1
H6N4 A/mallard/Sweden/81/2002	MNREFEVVNHEFSEVEKRINMINDKIDDQIEDLWTYNAELLVLLENERTLDLHDANVKNLYEKVKSQLRDNA	25	65.3
H11N9 A/shoveler/Netherlands/18/1999	MNTNFESVQHEFSEIEERINQLSKHVDDSVVDIWSYNAQLLVWLENEKTLDLHDSNVRNLHEKVRRMLKDNA	25	65.3
H8N4 A/mallard/Sweden/24/2002	MNREFEVVNHEFSEVEKRINMINDKIDDQIEDLWAYNAELLVLLENQKTLDEHDSNVKNLFDEVRRRLSANA	29	59.7
H9N2 A/guinea fowl/Hong Kong/WF10/1999	MNKQYEIIDHEFSEVETRLNMINNKIDDQIQDVWAYNAELLVLLENQKTLDEHDANVNNLYNKVKRALGSNA	33	54.2
H10N8 A/Jiangxi-Donghu/346/2013	TNTEFESIESEFSEIEHQIGNVINWTKDSITDIWTYQAELLVAMENQHTIDMADSEMLNLYERVRKQLRQNA	35	51.4
H4N6 A/red knot/Delaware/541/1988	TNEKYHQIEKEFEQVEGRIQDLEKYVEDTKIDLWSYNAELLVALENQHTIDVTDSEMNKLFERVRRQLRENA	37	48.6
H3N2 A/Wyoming/3/2003	TNEKFHQIEKEFSEVEGRIQDLEKYVEDTKIDLWSYNAELLVALENQHTIDLTDSEMNKLFERTKKQLRENA	38	47.2
H7N9 A/Shanghai/1/2013	TNQQFELIDNEFTEVEKQIGNVINWTRDSITEVWSYNAELLVAMENQHTIDLADSEMDKLYERVKRQLRENA	40	44.4

Note: 1—Number of mutations within the N-terminally extended long alfa helix region; 2—% identity.

Table S3. Sequence conservation between the vaccination antigens and the corresponding protein fragments of influenza A challenge viruses used in this study.

Reference Virus	Amino Acid Sequence	1	2
**tri-stalk antigen:**	MNTQFTAVGKEFNHLEKRIENLNKKVDDGFLDIWTYNAELLVLLENERTLDYHDSNVKNLYEKVRSQLKNNA		
H1N1 A/California/7/2009	MNTQFTAVGKEFNHLEKRIENLNKKVDDGFLDIWTYNAELLVLLENERTLDYHDSNVKNLYEKVRSQLKNNA	0	100
H1N1 A/Puerto Rico/8/1934	MNIQFTAVGKEFNKLEKRMENLNKKVDDGFLDIWTYNAELLVLLENERTLDFHDSNVKNLYEKVKSQLKNNA	5	93.1
H5N1 A/Viet Nam/1203/2004	MNTQFEAVGREFNNLERRIENLNKKMEDGFLDVWTYNAELLVLMENERTLDFHDSNVKNLYDKVRLQLRDNA	13	81.9
H3N2 A/Philippines/2/1982 (X-79)	TNEKFHQIEKEFSEVEGRIQDLEKYVEDTKIDLWSYNAELLVALENQHTIDLTDSEMNKLFEKTRKQLRENA	36	50
**3M2e antigen, fragment 1:**	MSLLTEVETPIRNEWGSRSNGSSD		
H1N1 A/Puerto Rico/8/1934	MSLLTEVETPIRNEWGCRCNGSSD	2	91.7
H3N2 A/Philippines/2/1982 (X-79)	MSLLTEVETPIRNEWGCRCNGSSD	2	91.7
H5N1 A/Viet Nam/1203/2004	MSLLTEVETPTRNEWECRCSDSSD	6	75
H1N1 A/California/7/2009	MSLLTEVETPTRSEWECRCSDSSD	7	70.8
**3M2e antigen, fragment 2:**	MSLLTEVETPTRNGWGSKSNGSSD		
H1N1 A/Puerto Rico/8/1934	MSLLTEVETPIRNEWGCRCNGSSD	5	79.2
H3N2 A/Philippines/2/1982 (X-79)	MSLLTEVETPIRNEWGCRCNGSSD	5	79.2
H5N1 A/Viet Nam/1203/2004	MSLLTEVETPTRNEWECRCSDSSD	7	70.8
H1N1 A/California/7/2009	MSLLTEVETPTRSEWECRCSDSSD	8	66.7
**3M2e antigen, fragment 3:**	MSLLTEVETPTRNEWESRSSGSSD		
H5N1 A/Viet Nam/1203/2004	MSLLTEVETPTRNEWECRCSDSSD	3	87.5
H1N1 A/California/7/2009	MSLLTEVETPTRSEWECRCSDSSD	4	83.3
H1N1 A/Puerto Rico/8/1934	MSLLTEVETPIRNEWGCRCNGSSD	5	79.2
H3N2 A/Philippines/2/1982 (X-79)	MSLLTEVETPIRNEWGCRCNGSSD	5	79.2

Note: 1—Number of mutations ; 2—% identity.
