# Supplementary material for: Construction and Immunogenicity of a Novel Multivalent Vaccine Prototype Based on Conserved Influenza Virus Antigens
Source: Vaccines (Basel). 2020 Apr 24;8(2):197. doi: 10.3390/vaccines8020197 (PMC7349063; doi:10.3390/vaccines8020197)
Supplement: Supplementary file 1 [file vaccines-08-00197-s001.zip › Supplementary Figures.pdf]

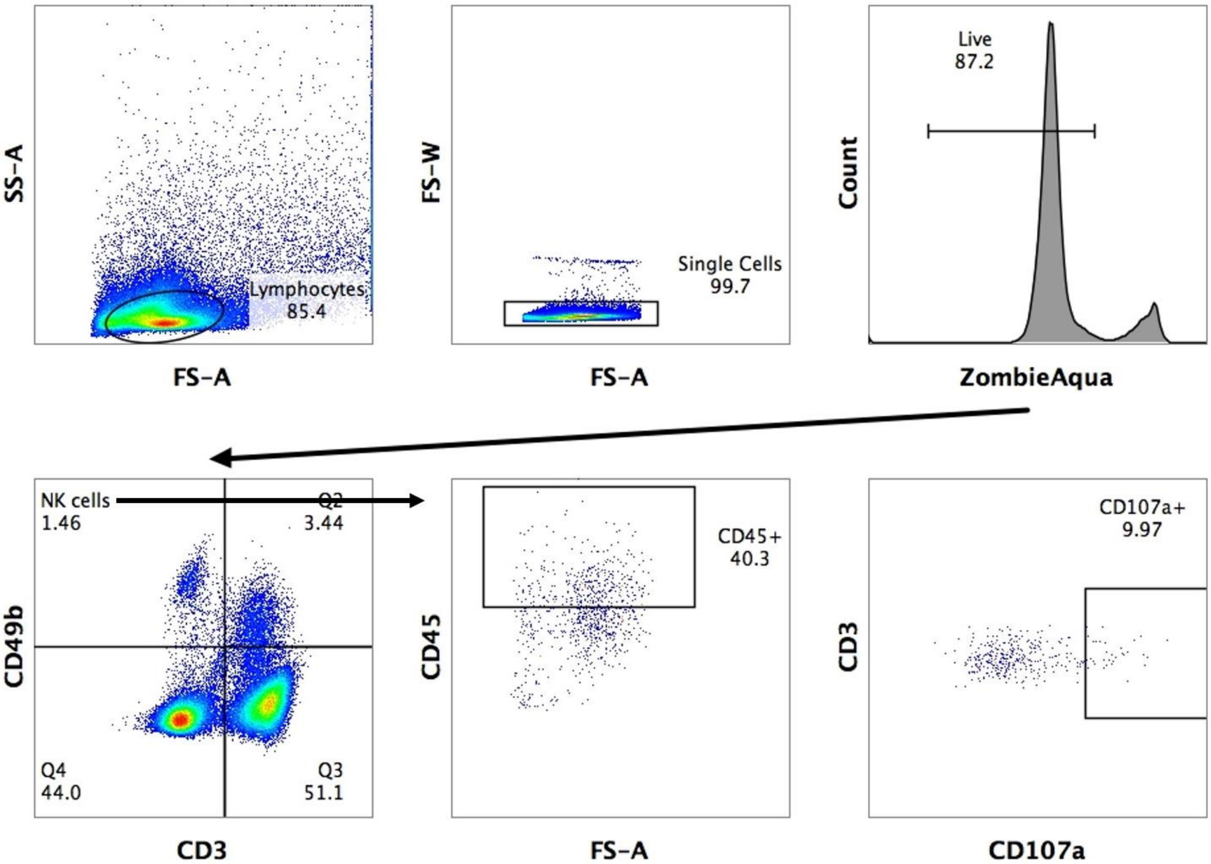

Figure S1. Gating strategy for NK-cell degranulation assay (antibody-dependent cellular cytotoxicity, ADCC).

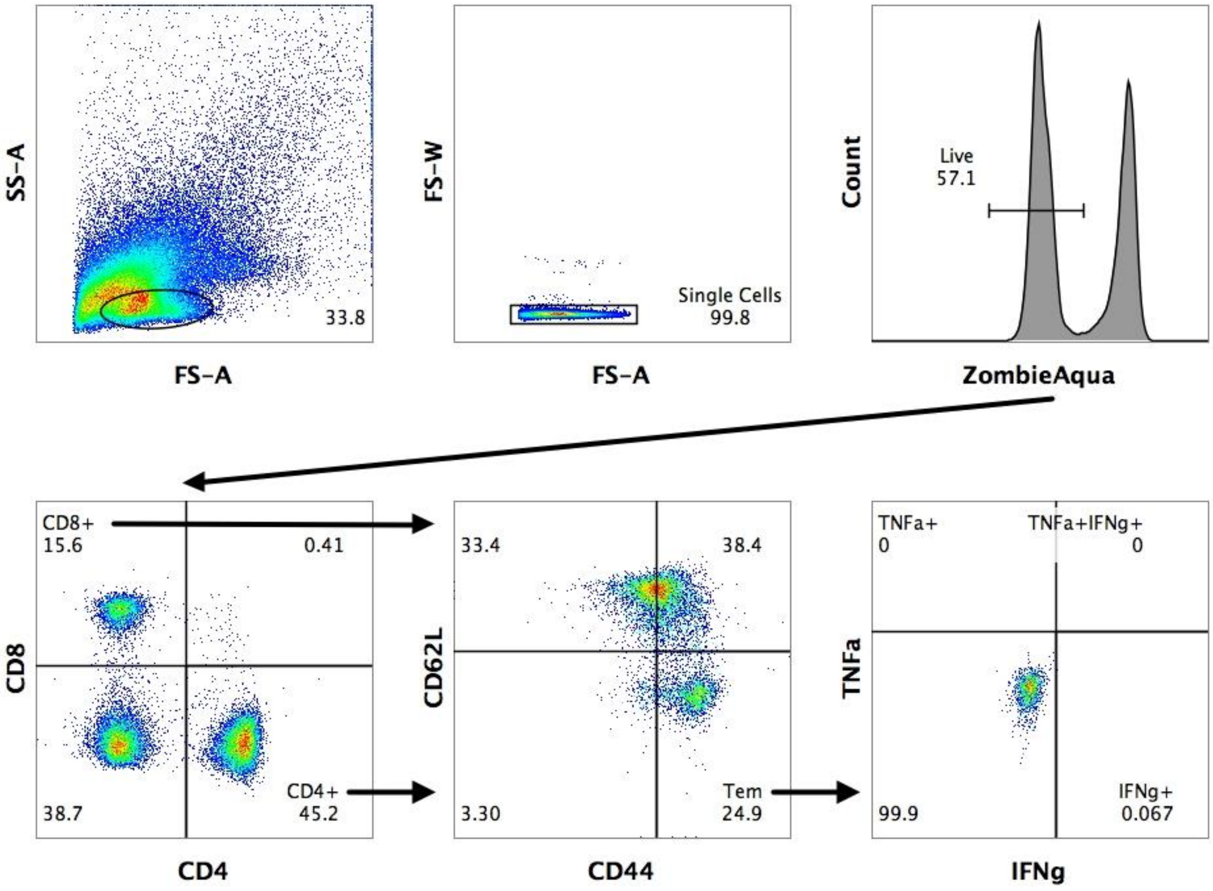

Figure S2. Gating strategy for intracellular cytokine staining (ICS) assay.

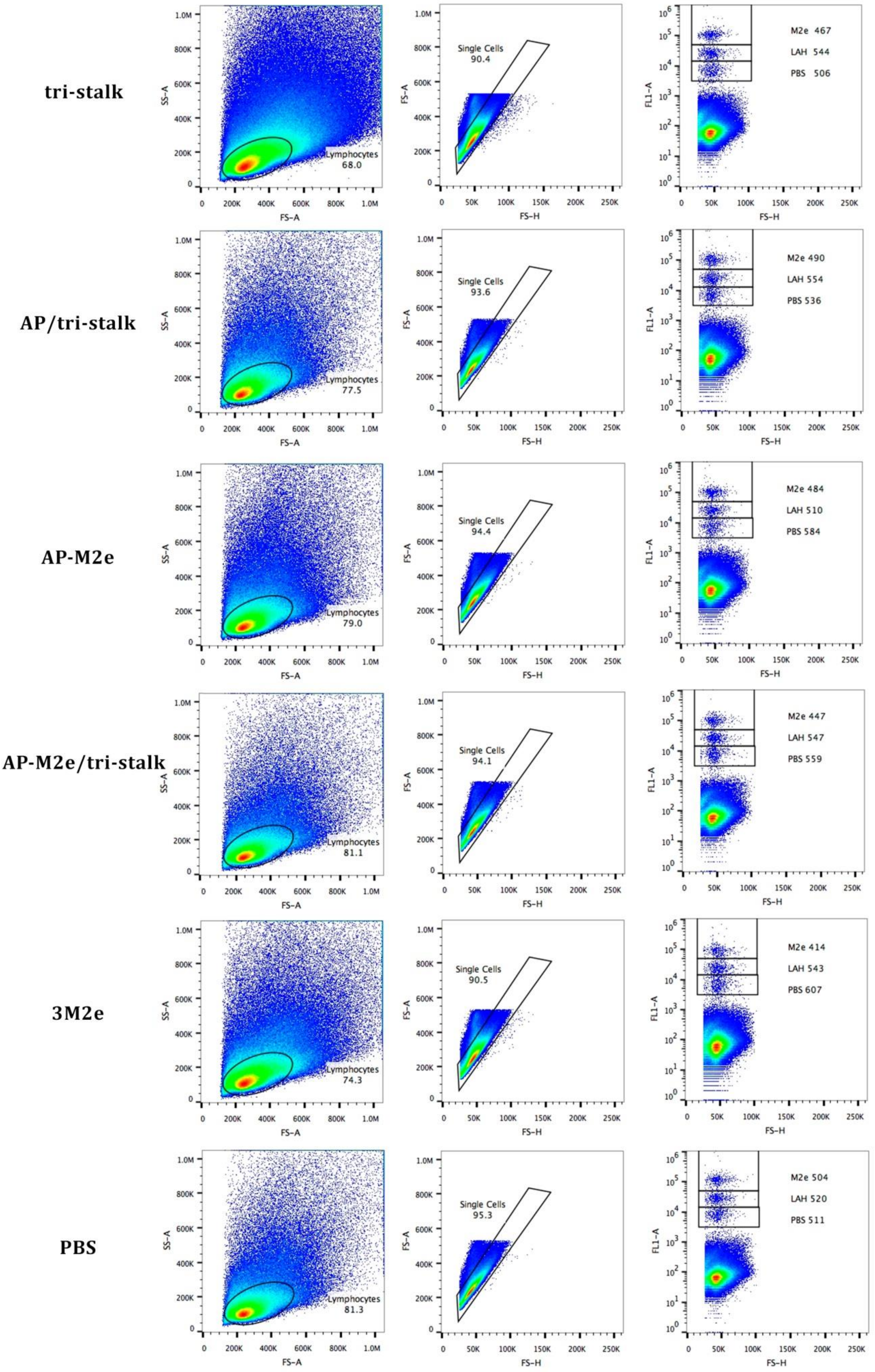

Figure S3. Gating strategy in flow cytometry analysis of target cells for CTL *in vivo* assay.
